# Structural and functional insights into thermally stable cytochrome *c*′ from a thermophile

**DOI:** 10.1002/pro.3120

**Published:** 2017-03-06

**Authors:** Sotaro Fujii, Hiroya Oki, Kazuki Kawahara, Daisuke Yamane, Masaru Yamanaka, Takahiro Maruno, Yuji Kobayashi, Misa Masanari, Satoshi Wakai, Hirofumi Nishihara, Tadayasu Ohkubo, Yoshihiro Sambongi

**Affiliations:** ^1^ Graduate School of Biosphere ScienceHiroshima UniversityHigashi‐HiroshimaHiroshimaJapan; ^2^ Graduate School of Pharmaceutical SciencesOsaka University, YamadaokaSuitaOsakaJapan; ^3^ Graduate School of Materials ScienceNara Institute of Science and TechnologyIkomaNaraJapan; ^4^ Graduate School of EngineeringOsaka University, YamadaokaSuitaOsakaJapan; ^5^ Graduate School of ScienceTechnology, and Innovation, Kobe University, RokkodaiKobeHyogoJapan; ^6^ Faculty of AgricultureIbaraki UniversityInashikigunIbarakiJapan

**Keywords:** protein stability, cytochrome c, thermophile, protein structure, mutagenesis, ligand‐binding

## Abstract

Thermophilic *Hydrogenophilus thermoluteolus* cytochrome *c*′ (PHCP) exhibits higher thermal stability than a mesophilic counterpart, *Allochromatium vinosum* cytochrome *c*′ (AVCP), which has a homo‐dimeric structure and ligand‐binding ability. To understand the thermal stability mechanism and ligand‐binding ability of the thermally stable PHCP protein, the crystal structure of PHCP was first determined. It formed a homo‐dimeric structure, the main chain root mean square deviation (rmsd) value between PHCP and AVCP being 0.65 Å. In the PHCP structure, six specific residues appeared to strengthen the heme‐related and subunit–subunit interactions, which were not conserved in the AVCP structure. PHCP variants having altered subunit–subunit interactions were more severely destabilized than ones having altered heme‐related interactions. The PHCP structure further revealed a ligand‐binding channel and a penta‐coordinated heme, as observed in the AVCP protein. A spectroscopic study clearly showed that some ligands were bound to the PHCP protein. It is concluded that the dimeric PHCP from the thermophile is effectively stabilized through heme‐related and subunit–subunit interactions with conservation of the ligand‐binding ability.

**Brief Summary:**

We report the X‐ray crystal structure of cytochrome *c*′ (PHCP) from thermophilic *Hydrogenophilus thermoluteolus*. The high thermal stability of PHCP was attributed to heme‐related and subunit–subunit interactions, which were confirmed by a mutagenesis study. The ligand‐binding ability of PHCP was examined by spectrophotometry. PHCP acquired the thermal stability with conservation of the ligand‐binding ability. This study furthers the understanding of the stability and function of cytochromes *c*.

## Abstract 2

PDB Code(s): 5B3I

**Abbreviations**
AVCP: *Allochromatium vinosum cytochrome c′*
CD: circular dichroism
PAGE: polyacrylamide gel electrophoresis
PHCP: *Hydrogenophilus thermoluteolus cytochrome c'*

## Introduction

Cytochromes *c*′ are class II cytochrome *c* proteins found in the periplasm of a variety of Gram‐negative bacteria.^1^, ^2^ The function of cytochromes *c*′ *in vivo* is unclear, but some of their physiological roles relate to cellular defense against NO stress.^3^ The cytochrome *c*′ proteins typically form a homo‐dimeric structure, and each single subunit forms a four‐helix bundle structure with a penta‐coordinate heme.^4^

Previously, we found that cytochrome *c*′ (PHCP) from thermophilic *Hydrogenophilus thermoluteolus* (formally called *Pseudmonas hydrogenothermophila*) growing optimally at 52°C showed high thermal stability compared with cytochrome *c*′ (AVCP) from mesophilic *Allochromatium vinosum* growing optimally at 25–30°C.^5^, ^6^, ^7^ Cytochrome *c*′ from another mesophilic bacterium, *Shewanella amazonensis*, growing optimally at 37°C exhibited thermal stability that was intermediate between those of AVCP and PHCP.^8^ The thermal stability of these three proteins positively correlates with the growth temperatures of the source bacteria, thus a comparative study on protein stability can be carried out using these proteins.

Thioether bond formation between two vinyl groups of a heme and two Cys residues in the Cys‐Xaa‐Xaa‐Cys‐His motif, which chemically defines a cytochrome *c* protein, is prerequisite for the stability of PHCP,^9^ as discussed previously for class I monomeric globular cytochromes *c*.^10^ The stability of class I cytochromes *c* from some thermophilic bacteria is further enhanced by noncovalent interactions between the heme and amino acid side chains in addition to the thioether bond formation, as compared with that of homologous counterparts from mesophiles.^11^, ^12^, ^13^, ^14^, ^15^, ^16^, ^17^ The stability of thermophilic class I cytochromes *c* is attributed to the densely packed heme pocket with conservation of the biological function, i.e., acquisition and release of electrons.

The functional situation should differ between class I and II cytochromes *c*. Class II cytochromes *c* bind gas molecules through the heme *in vitro*, as extensively revealed using cytochrome *c*′ (AXCP) from mesophilic *Alcaligenes xylosoxidans*.^18^ In AXCP, NO binds to the fifth coordinate position of the heme, at which the His residue in the Cys‐Xaa‐Xaa‐Cys‐His motif is used for coordination, after forming sixth coordinate NO intermediate and dinitrosyl species.^19^ This gas‐binding ability is mainly regulated by an Arg residue around the fifth position.^20^ Furthermore, O_2_ binds to the sixth coordinate position of the heme when the original Leu residue is replaced by Ala around the sixth position.^21^ Thus the gas‐binding ability of the AXCP protein is controlled by some specific amino acid residues around the heme.

It is unknown whether or not PHCP binds gas molecules like AXCP does and whether it has functional residues for gas‐binding in the three‐dimensional structure. In addition, AVCP has a channel structure, which allows the access of gas molecules and ligands such as alkyl‐isocyanides or imidazole to the heme.^22^ If PHCP has such a channel structure, it is also unknown how the thermal stability and the ligand‐binding ability are related, densely packed protein folding and enough space around the heme possibly being required, respectively, which do not seem to be compatible.

Here we first determined the crystal structure of thermally stable PHCP, which exhibited some potential amino acid residues responsible for the high thermal stability and a conserved channel for the ligand‐binding ability. In order to confirm these structural predictions, we carried out a mutagenesis study on the PHCP protein for the thermal stability mechanism and visible spectroscopy measurement for the ligand‐binding ability. Based on the results obtained, the stability mechanism of the PHCP protein in conjunction with the ligand‐binding ability is discussed.

## Results and Discussion

### Overall structure

**Figure 1:**
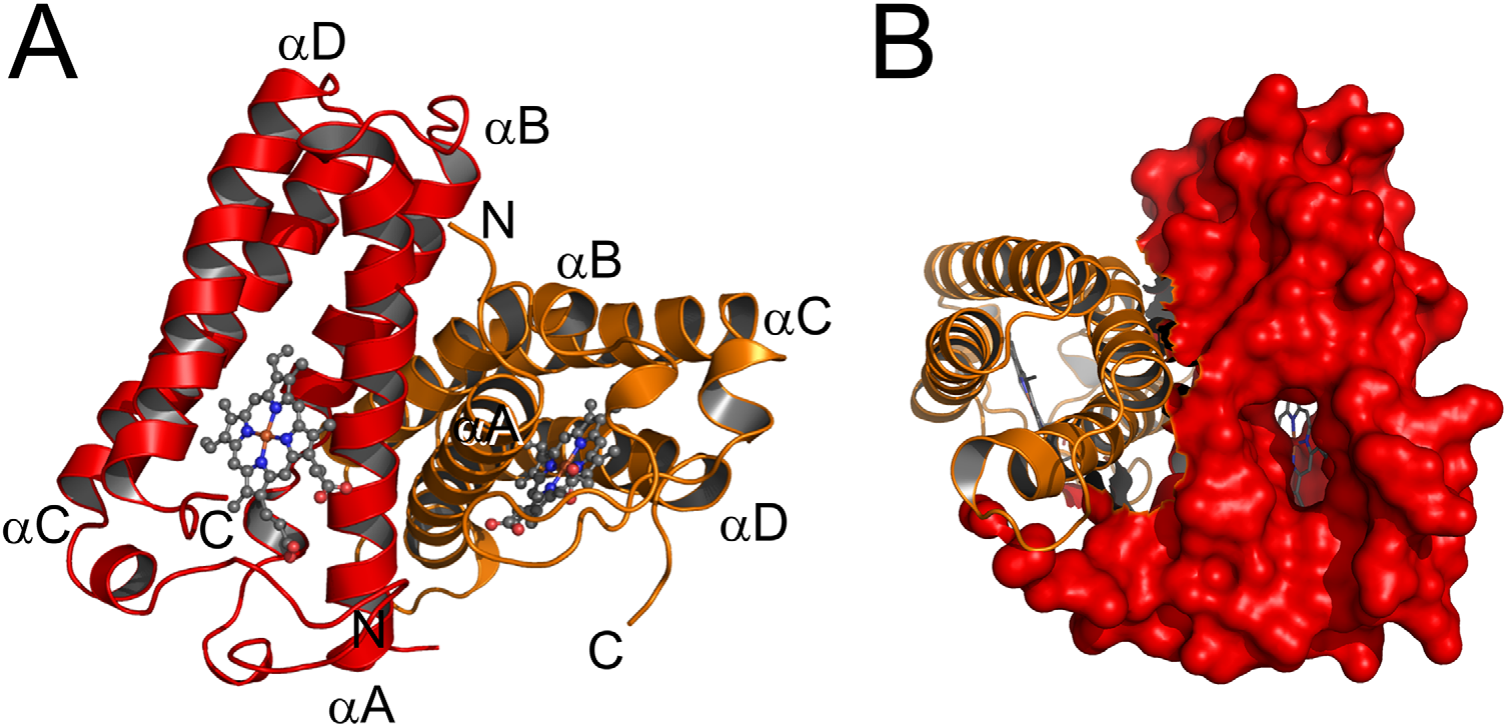
A. Overall dimeric structure of PHCP. The main chain and heme are presented as ribbon and stick models, respectively. The single subunits are colored red and orange. The N‐ and C‐ termini as well as helical regions in each subunit are indicated. B. A channel to the sixth coordinate position of the heme for the ligand‐binding ability in PHCP. A single subunit of PHCP is shown with a red surface. The heme is shown as a stick model.

The overall crystal structure of the PHCP protein was determined by the molecular replacement method using the AVCP crystal structure as a search model, and was refined to 1.89 Å resolution. The asymmetric unit of the crystal contained two copies of the PHCP homo‐dimer (Supporting Information Fig. S1), which superimposed well on each other, the rmsd value being 0.31 Å, when the backbone Cα atoms were used. The PHCP dimeric structure [Fig. 1(A)] resembled that of AVCP. The PHCP dimeric structure was also consistent with the result obtained on ultracentrifugation analysis in this study described below.

**Figure 2:**
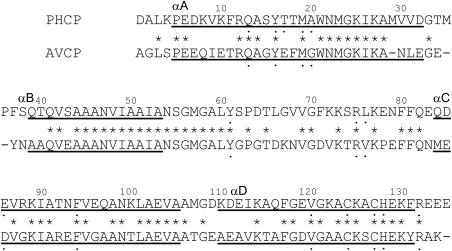
Amino acid sequences of PHCP and AVCP. Gaps in the sequences, identical residues, and α‐helical regions of PHCP (this study) and AVCP^22^, ^43^ are indicated by dashes, asterisks, and underbars, respectively. Dotted residues are those located near the heme within 4.0 Å.

Although the PHCP protein clearly forms a dimeric structure, as shown above, previous gel filtration and blue native PAGE experiments indicated a trimeric structure.^6^ A similar discrepancy in molecular mass estimation often occurs on gel filtration analysis.^23^ Furthermore, the PHCP sequence contains three successive Glu residues at the C‐terminus (Fig. 2), which may not bind the negatively charged dye molecules used for the blue native PAGE experiments, thus resulting in wrong estimation of the molecular mass, as previously observed.^24^

The single subunit of PHCP formed a four‐helix bundle structure [Fig. 1(A)]. The main chains of each subunit in the dimeric PHCP protein superimposed well on each other, the rmsd value being 0.20 Å. A single subunit consisted of four α‐helices, αA (Pro‐5 to Asp‐32), αB (Gln‐39 to Ala‐53), αC (Gln‐85 to Ala‐105), and αD (Lys‐110 to Phe‐131). Four α‐helices were also conserved in the AVCP protein (Fig. 2). The main chain structure of the single PHCP subunit superimposed well on that of AVCP, the rmsd value between the Cα atoms being 0.65 Å.

A channel structure with dimensions of 8.9 × 13.8 Å possibly for ligand‐binding to the heme was observed in PHCP [Fig. 1(B)]. This channel was surrounded mainly by hydrophobic residues (Ile‐52, Ala‐53, Ser‐55, Gly‐56, Gly‐58, Tyr‐61, Phe‐80, Gln‐84, Val‐87, Ala‐91, and Phe‐94) on the αB and αC helices. The AVCP protein, having ligand‐binding ability, exhibited a similar channel with dimensions of 7.5 × 11.8 Å, as discussed previously.^22^ It should be noted that the amino acid residues forming the AVCP channel structure were all conserved in PHCP with the exception of that at position 84 (Fig. 2). Judging from the overall structure of PHCP, the stability difference between PHCP and AVCP should be mostly attributed to differences in the side chains of amino acids residues not forming the channel structure.

### Heme‐related interactions

**Figure 3:**
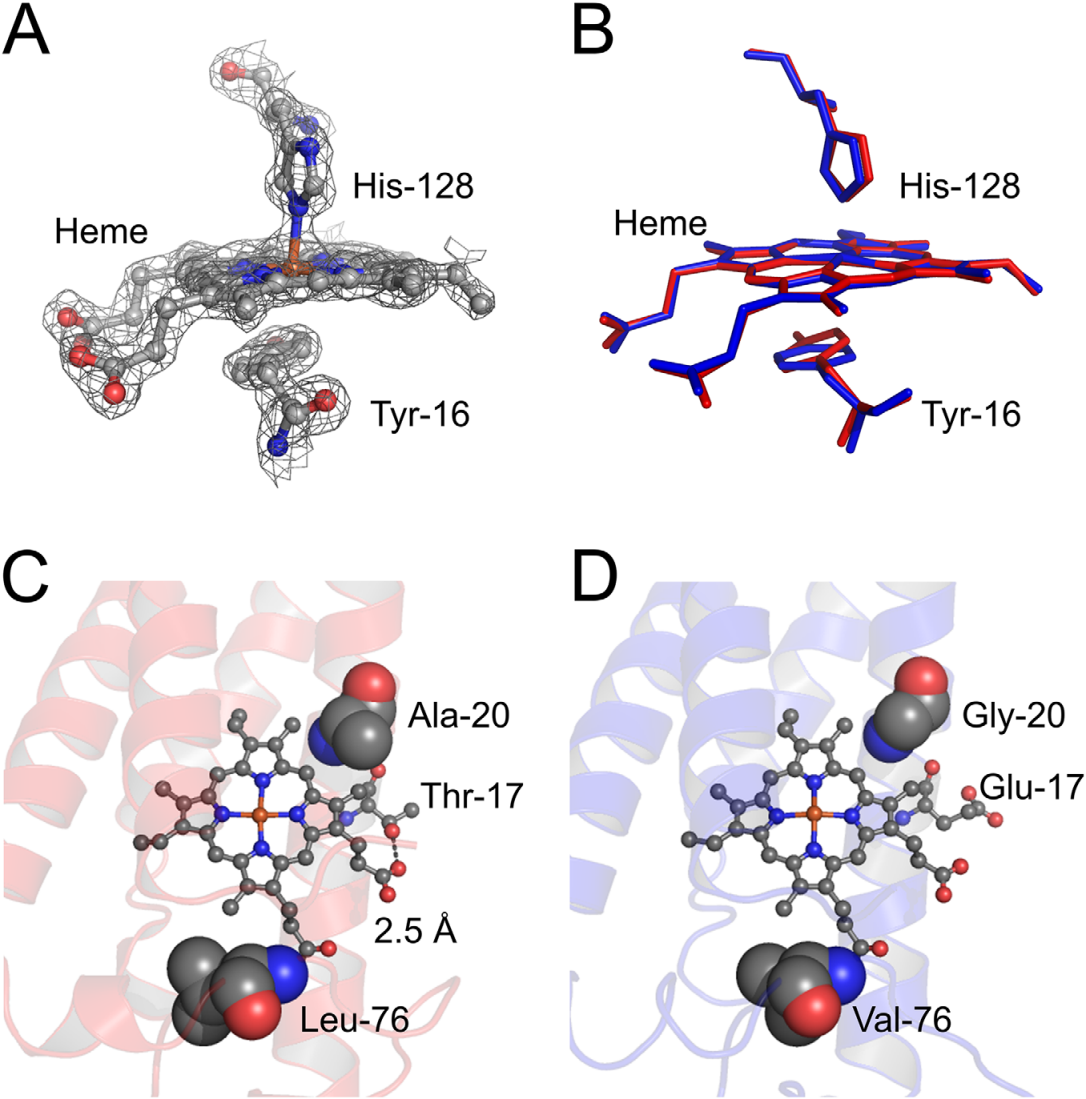
A. 2m*F*_o_‐D*F*_c_ electron density map of penta‐coordinate heme, contoured at 1.5σ. The side chain and the heme are presented as ball‐and‐stick model. B. Heme environment of PHCP (red) and AVCP (blue). The side chain and heme are shown as a stick model. C. Heme‐related interactions in PHCP. The heme and relevant amino acid residues are presented as ball‐and‐stick models. The hydrogen bond is shown by a black dotted line. Only one of the two subunits in PHCP is shown. D. Heme‐related interactions in AVCP.

In a single subunit of the PHCP structure, the side chain atoms of 15 amino acid residues, Gln‐13, Tyr‐16, Thr‐17, Met‐19, Ala‐20, Tyr‐61, Arg‐75, Leu‐76, Val‐87, Phe‐94, Val‐120, Cys‐124, Cys‐127, His‐128, and Arg‐132, were located around the heme atoms within 4.0 Å (Fig. 2), and most of these 15 residues were conserved in AVCP. Among them, Cys‐124 and Cys‐127 were covalently bound with the heme through thioether linkages. His‐128 was coordinated to the fifth position of the proximal site of the heme iron, and Tyr‐16 was located at the vacant sixth coordinate position parallel with the heme [Fig. 3(A)]. The penta‐coordinate heme, Tyr‐16 and His‐128 of PHCP superimposed well on those of AVCP [Fig. 3(B)]. However, Thr‐17, Ala‐20, and Leu‐76 of the PHCP protein were substituted by Glu, Gly, and Val in AVCP, respectively [Figs. 2, 3(C, D)]. Thr‐17 and Ala‐20 were on the αA helix, and Leu‐76 was in the longest loop between the αB and αC helices (Fig. 2).

The PHCP Thr‐17 O1A atom formed a hydrogen bond with the heme 17‐propionate OG1 atom in 2.5 Å [Fig. 3(C)], whereas the corresponding AVCP Glu‐17 side chain protruded from the protein surface without any interaction with the heme [Fig. 3(D)]. PHCP Ala‐20 and Leu‐76 hydrophobically interacted with the heme more favorably than the corresponding AVCP Gly‐20 and Val‐76 [Fig. 3(C, D)], because these PHCP residues each carried an additional methyl group compared with the respective AVCP ones, and thus the heme environment was more densely packed in the PHCP protein. The residues at these three positions may be responsible for the difference in stability between PHCP and AVCP through the noncovalent interactions between the heme and amino acid side chains. Stabilization through heme‐related interactions is also observed in class I monomeric cytochromes *c* from thermophiles.^11^, ^12^, ^13^, ^14^, ^15^, ^16^, ^17^

### Subunit–subunit interactions

As to the stability difference between PHCP and AVCP, we further investigated side chain interactions on the subunit–subunit interface. The interface area of PHCP was 1073.5 Å^2^, which was calculated as the sum of the surface areas of amino acid residues that formed the subunit–subunit interface with a probe radius of 1.4 Å using the PyMOL program, whereas that in AVCP was 704.3 Å^2^. This indicated that PHCP had a wider interaction area between the two subunits than AVCP. This area difference was because of the presence of 11 specific amino acid residues in PHCP, Asp‐1, Phe‐11, Gln‐13, Ser‐15, Thr‐17, Thr‐18, Ala‐20, Thr‐34, Met‐35, Phe‐71, and Lys‐72, which were not conserved in AVCP [Supporting Information Fig. S2(A, B)].

**Figure 4:**
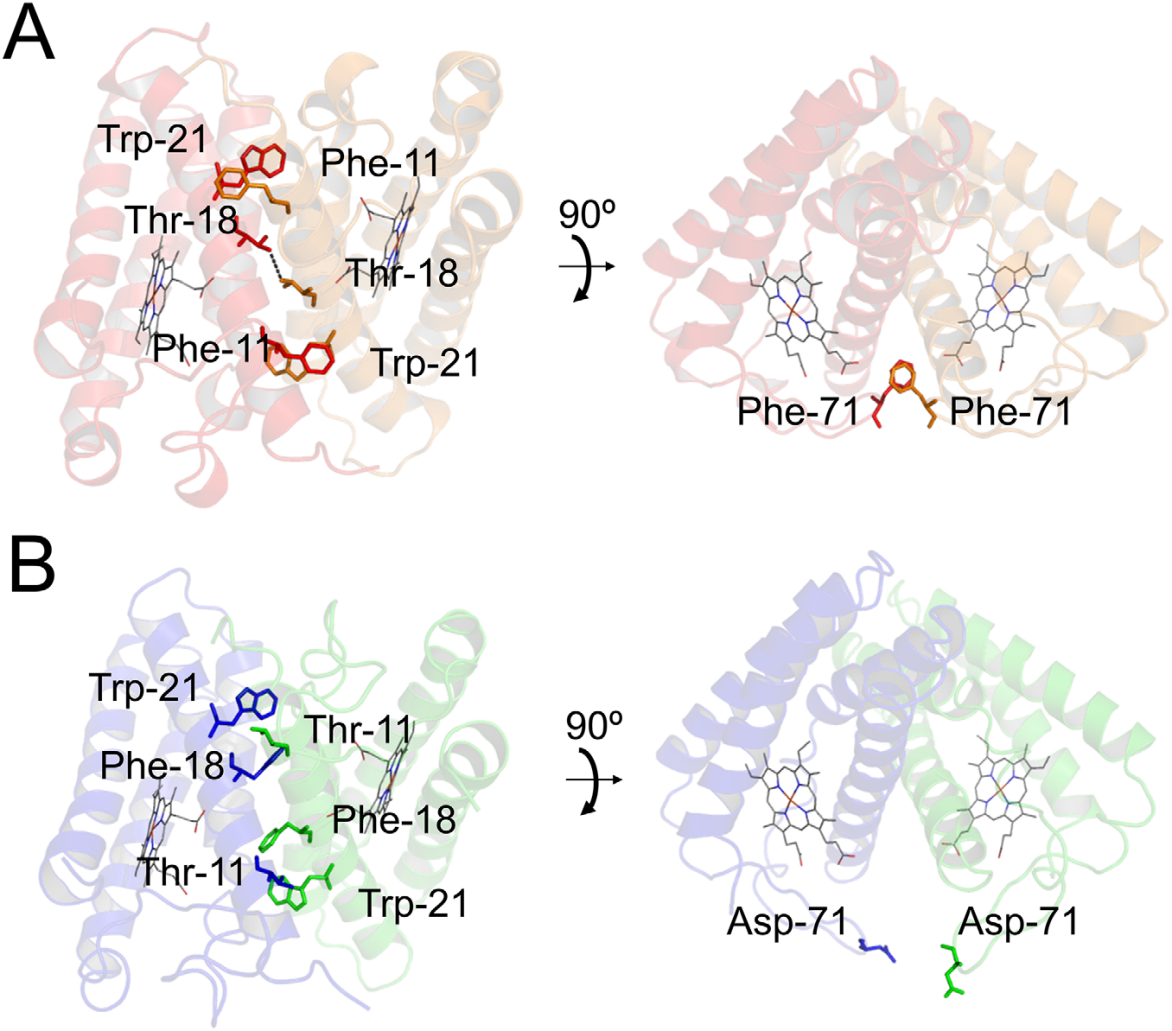
Differences between PHCP and AVCP on the subunit–subunit interface. A. PHCP structure. B. AVCP structure. The main chains and hemes are presented as ribbon and line models, respectively. Specific amino acid residues are presented as a stick model. The hydrogen bond is shown by a black dotted line.

Among the 11 specific residues in PHCP, Phe‐11 in the αA helix was located close to Trp‐21 in the αA helix of the other subunit [Fig. 4(A)], and hydrophobically filled a gap between the subunits. The corresponding hydrophilic Thr‐11 in AVCP [Fig. 4(B)] may destabilize the subunit–subunit interaction. The two Thr‐18 side chains on the αA helices of the two PHCP subunits formed a hydrogen bond [Fig. 4(A)]. In contrast, the corresponding Phe‐18 residues in AVCP did not form such a hydrogen bond [Fig. 4(B)]. Two Phe‐71 residues in the PHCP subunits formed a π−π stacking structure in the loops between the αB and αC helices, which was unique to the PHCP subunit–subunit interface [Fig. 4(A)]. The corresponding Asp‐71 residues in AVCP may cause electrostatic repulsion [Fig. 4(B)]. The other eight amino acid residues on the PHCP subunit–subunit interface did not appear to undergo specific interaction like that observed for Phe‐11, Thr‐18, and Phe‐71.

It has been stated that two subunits of mesophilic cytochromes *c*′ interact mainly at the αA and αB helices,^25^ which is the case in PHCP [Fig. 1(A)] and AVCP. Our present findings showed that the PHCP protein exhibited specific interactions involving by Phe‐11, Thr‐18, and Phe‐71 on the subunit–subunit interface compared with the AVCP protein. These specific interactions between the two subunits may be responsible for the high stability of PHCP, as compared with that of the AVCP protein.

In other dimeric proteins from a thermophile, additional interactions on the subunit–subunit interfaces compared with those in counterparts from a mesophile have also been observed: Phosphoribosyl anthranilate isomerase and 3‐isopropylmalate dehydrogenase of *Thermus thermophilus* showed high stability because of subunit–subunit interactions compared with the counterparts from mesophilic *E. coli*.^26^, ^27^, ^28^ In conjunction with the present findings for the PHCP protein, specific amino acid residues located on the subunit–subunit interfaces of dimeric proteins from thermophiles may be responsible for the stability.

### Mutagenesis and thermal stability measurement

On the three‐dimensional structure analysis of PHCP described above, six residues (Phe‐11, Thr‐17, Thr‐18, Ala‐20, Phe‐71, and Leu‐76) were suggested to be responsible for the stability, as compared with AVCP. Thus we examined the contributions of these residues to the stability of the PHCP protein through a mutagenesis study by replacement of the corresponding amino acid residues originally found in the AVCP protein.

**Figure 5:**
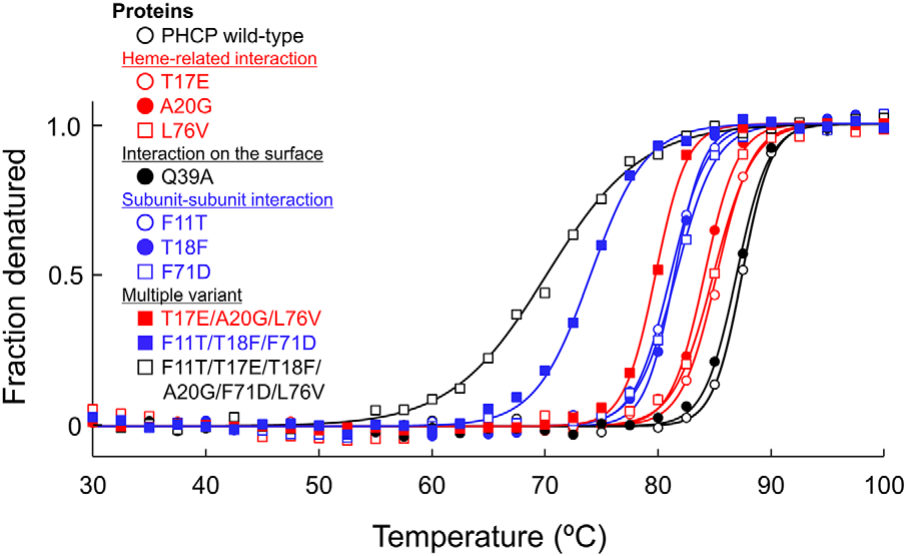
Thermal denaturation, as measured by CD. Representative normalized raw data points are shown at a temperature interval of 2.5°C. Fitting curves for each data set are also shown.

Thermal denaturation curves obtained on circular dichroism (CD) measurement for the recombinant PHCP wild‐type and its variant proteins were normalized, as shown in Figure 5. All these denaturation curves were S‐shaped, indicating a two‐state denaturation transition. After completion of the heating process up to each *T*_m_, the protein solutions were cooled and then kept at 30°C. From the CD spectra obtained at 30°C before and after the heating, it was confirmed that 98% of the native state was retained (data not shown), suggesting reversible thermal denaturation processes. These results indicated that the thermal denaturation observed in the present study correctly provided equilibrium thermodynamic parameters (Table 1).

**Table 1:** Parameters Characterizing Thermal Denaturation Obtained on CD Measurement for the Recombinant PHCP Wild‐Type and its Variant Proteins

Protein	*T*_m_ (°C)	Δ*T*_m_ (°C)	ΔΔ*G*_m_ (kJ/mol)
PHCP wild‐type	87.4 ± 0.1	0	0
**Heme‐related interaction**			
T17E	85.4 ± 0.2	−2.0	−4.3 ± 0.4
A20G	83.8 ± 0.6	−3.6	−7.7 ± 1.2
L76V	84.8 ± 0.2	−2.6	−5.5 ± 0.5
**Interaction on the surface**			
Q39A	87.1 ± 0.2	−0.3	−0.6 ± 0.4
**Subunit–subunit interaction**			
F11T	81.1 ± 0.2	−6.4	−13.7 ± 0.3
T18F	81.6 ± 0.2	−5.8	−12.3 ± 0.5
F71D	81.8 ± 0.2	−5.6	−12.1 ± 0.3
**Multiple variant**			
T17E/A20G/L76V	79.5 ± 0.3	−7.9	−16.8 ± 0.8
F11T/T18F/F71D	74.3 ± 0.4	−13.1	−28.1 ± 0.8
T11F/T17E/T18F/A20G/F71D/L76V	70.0 ± 0.3	−17.4	−37.2 ± 0.6

The *T*_m_ and ΔΔ*G*_m_ values presented are means ± SD for triplicate experiments. The Δ*T*_m_ and ΔΔ*G*_m_ values are ones calculated taking those of the PHCP wild‐type as zero.

The *T*_m_ value of the present recombinant PHCP wild‐type protein was 87.4°C (Table 1), which was the same as that of the authentic form,^6^ indicating that the recombinant PHCP protein was formed in *E. coli* cells as it was in the original thermophilic *H. thermoluteolus* cells. Therefore, the pure effects of mutations on the stability of the PHCP protein could be evaluated using the recombinant variant proteins.

### Stability of variants having altered heme‐related interactions in a single subunit

The three PHCP single variants, having altered heme‐related interactions in a single subunit, i.e., T17E (Thr‐17 to Glu), A20G (Ala‐20 to Gly), and L76V (Leu‐76 to Val), exhibited lower *T*_m_ and ΔΔ*G*_m_ values than those of the wild‐type (Table 1), indicating that these single mutations caused destabilization in terms of thermal stability. Structure simulation analysis indicated that Gly‐20 and Val‐76 introduced caused a void space, and that Glu‐17 introduced did not form a hydrogen bond but caused steric hindrance with the heme propionic acid group [Supporting Information Fig. S3(A)]. The Glu‐17 side chain, however, may be able to form a salt bridge with the Lys‐10 side chain on the other subunit [Supporting Information Fig. S3(B)], possibly mitigating the destabilization effect. Thus, the absolute ΔΔ*G*_m_ value of the T17E variant was smaller than the theoretical one for a hydrogen bond (8–16 kJ/mol).

### Hydrogen bond on the single subunit surface

Gln‐39 at the beginning of the αB helix appeared to form a hydrogen bond with Ala‐105 at the end of the αC helix in the same subunit of the PHCP protein [Supporting Information Fig. S4(A)]. This interaction occurred on the single subunit surface and was not related to the heme. The corresponding Ala‐39 residue in the AVCP protein did not form a hydrogen bond with Ala‐105 [Supporting Information Fig. S4(B)]. The PHCP Q39A (Gln‐39 to Ala) variant exhibited almost the same *T*_m_ and ΔΔ*G*_m_ values as those of the PHCP wild‐type (Table 1), indicating that the hydrogen bond formed between Gln‐39 and Ala‐105 did not contribute to the overall protein stability. This finding emphasizes the present observation that heme‐related interactions, not those on the surface, in the same subunit play significant roles in the PHCP stability.

### Stability of variants having altered subunit–subunit interactions

The three PHCP single variants, having altered subunit–subunit interactions, i.e., F11T (Phe‐11 to Thr), T18F (Thr‐18 to Phe), and F71D (Phe‐71 to Asp), exhibited lower *T*_m_ and ΔΔ*G*_m_ values than those of the wild‐type (Table 1), indicating that these single mutations caused destabilization. Structure simulation analysis indicated that the hydrophilic Thr‐11 side chain introduced onto one subunit of PHCP did not hydrophobically interact with the hydrophobic Trp‐21 side chain on the other subunit [Supporting Information Fig. S5(A)]. The two Phe‐18 residues introduced into the two PHCP subunits may undergo a hydrophobic interaction with each other [Supporting Information Fig. S5(A)], which appeared to be less effective for the thermal stability than the original Thr‐18 residues that formed a hydrogen bond with each other, as discussed above (Fig. 4). The hydrophilic Asp‐71 side chains introduced may cause destabilization in a local hydrophobic environment [Supporting Information Fig. S5(B)].

### Comparison of single and multiple mutations

The six specific amino acid residues interacting with the heme in the same subunit (Thr‐17, Ala‐20, and Leu‐76) or with the other residues in the other subunit (Phe‐11, Thr‐18, and Phe‐71) contributed to the stability, as described above. The resulting ΔΔ*G*_m_ values obtained for the single variants having altered subunit–subunit interactions were significantly lower than those of the single variants having altered heme‐related interactions (Table 1).

The PHCP triple variant, having F11T/T18F/F71D mutations that altered subunit–subunit interactions at the same time, exhibited lower *T*_m_ and ΔΔ*G*_m_ values than those of the PHCP triple variant, having T17E/A20G/L76V mutations that altered heme‐related interactions in the single subunit (Table 1). These results were consistent with those for the single mutations described above, showing that the subunit–subunit interactions were more effective than the heme‐related ones.

The *T*_m_ and ΔΔ*G*_m_ values of the T11F/T17E/T18F/A20G/F71D/L76V sextuple variant were 70.0°C and −37.2 kJ/mol, which were significantly lower than those of the PHCP wild‐type and all other variants (Table 1). The reason for the prominent decrease in stability of the sextuple variant will be provided by results obtained on ultracentrifugation analysis in this study described below.

### Quality of recombinant proteins

In order to confirm the quality of variant proteins that should affect the thermal stability, the following analyses were performed on the recombinant PHCP wild‐type and its variants. The N‐terminal sequence of the recombinant PHCP wild‐type and its A20G variant was determined to be Asp‐Ala‐Leu‐Lys‐Pro, which was the same as that of the authentic mature PHCP protein.^29^ All other variant proteins exhibited the same band position on SDS‐PAGE, indicating that the signal peptides for these recombinant proteins were removed correctly in the *E. coli* cells.

The recombinant PHCP wild‐type and variant proteins all gave heme staining bands on SDS‐PAGE (data not shown). The visible spectra of the recombinant PHCP wild‐type protein in the oxidized and reduced states showed the same specific peaks as those of the authentic PHCP protein [Supporting Information Fig. S6(A, B)]. Also, all the single variants and the T17E/A20G/L76V triple variant also gave the same visible absorption spectra (350–700 nm) as those of the PHCP wild‐type in the oxidized and reduced states, indicating that the heme was correctly incorporated into these apo‐proteins and covalently bound to them in the *E. coli* cells. However, the specific visible absorption peaks of the F11T/T18F/F71D triple and F11T/T17E/T18F/A20G/F71D/L76V sextuple variants in the oxidized state shifted from the wild‐type 398 to 402 and 407 nm, respectively [Supporting Information Fig. S7(A)]. The reason for the spectral changes for these multiple variants may be a slight change in the heme environment. CD spectra (200–250 nm) of the F11T/T18F/F71D triple and the sextuple variants each exhibited a peak at around 222 nm [Supporting Information Fig. S7(B)]. Although the peak intensity of the sextuple variant was rather less than those of the wild‐type and the triple variants, this peak could be followed during the protein thermal denaturation experiments described above.

**Figure 6:**
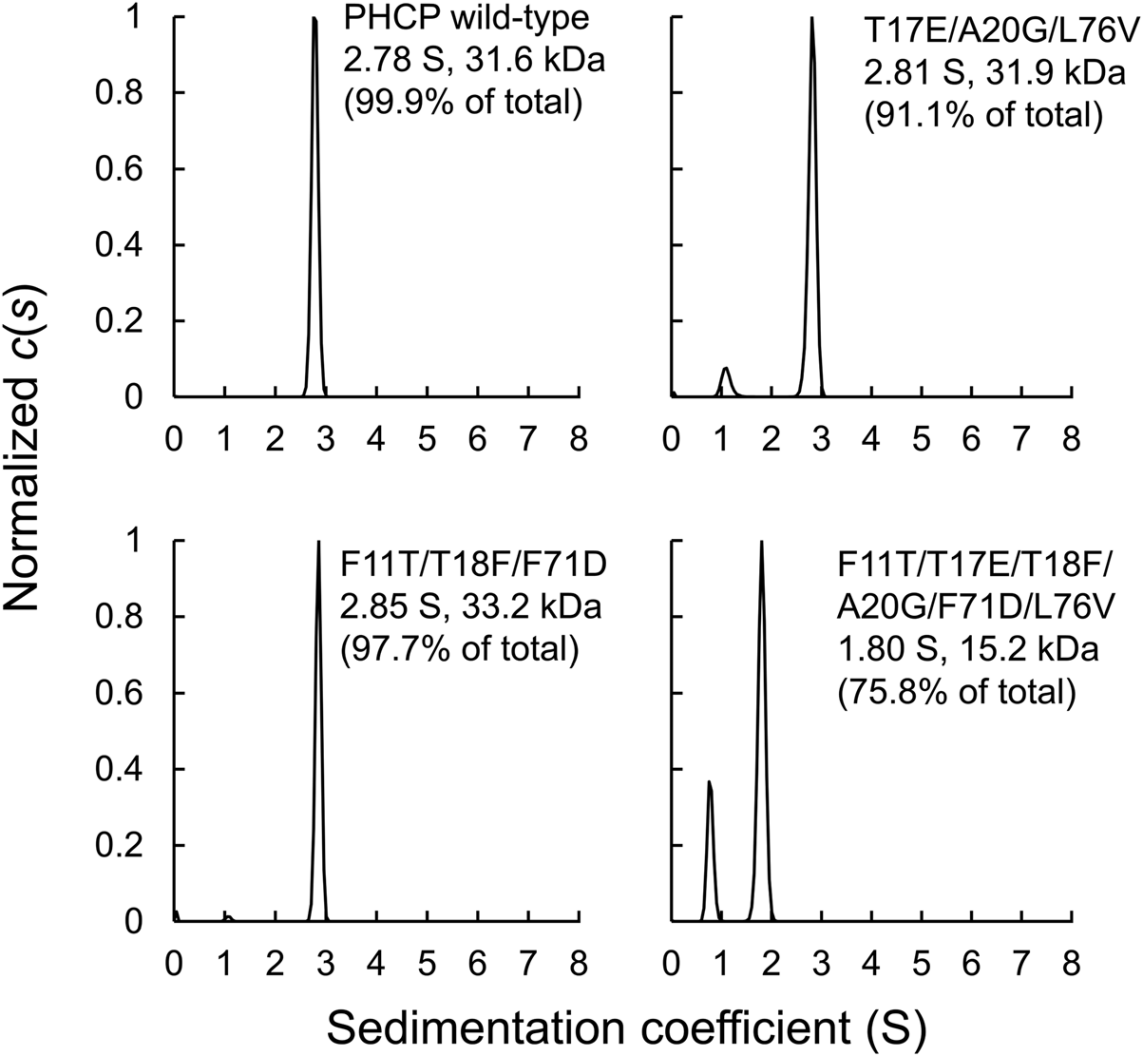
Sizes of the PHCP wild‐type and its variants determined by analytical ultracentrifugation (*n* = 1). The distributions of the sedimentation coefficients (S) of the PHCP wild‐type and its variants are shown. The data were analyzed using the continuous *c(s)* distribution model in the program SEDFIT.

The subunit compositions of the recombinant PHCP proteins were analyzed by means of sedimentation velocity experiments involving ultracentrifugation (Fig. 6). The PHCP wild‐type showed an S value of 2.78 S with a 99.9% population, the calculated mass being 31.6 kDa, which was in agreement with the dimeric structure determined on the crystal structure analysis described above. Although the dimer population was less than in the case of the wild‐type, the triple T17E/A20G/L76V variant with a population of 91.1% had the same S value as that of the wild‐type, indicating that three mutations together did not affect the dimer formation. The triple F11T/T18F/F71D variant with a 97.7% population also exhibited an S value of 2.85 S, which was an indication of a dimeric structure. The same analysis also indicated that the each single variant that exhibited lower stability than the wild‐type, i.e., T17E, A20G, L76V, F11T, T18F, and F71D, formed a dimer structure (data not shown).

In contrast, the sextuple F11T/T17E/T18F/A20G/F71D/L76V variant did not exhibit an S value corresponding to a dimeric mass, the values with 75.8 and 23.9% populations being 1.80 and 0.78 S, respectively (Fig. 6), and the former main population corresponded to 15.2 kDa, indicating that this variant mainly formed a monomeric structure. Therefore, the prominent decrease in the stability of the sextuple variant may be related to dimer dissociation.

### Ligand‐binding assay

**Figure 7:**
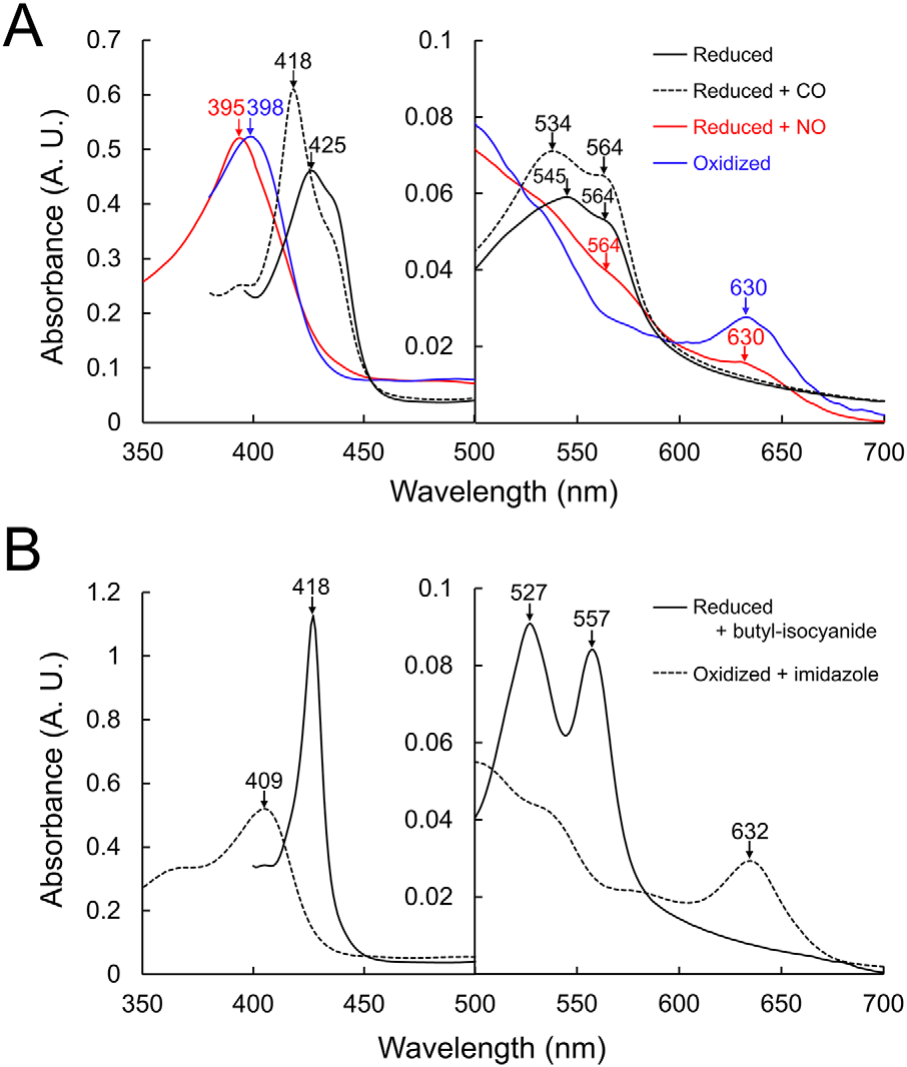
A. Visible absorption spectra of the reduced PHCP wild‐type protein with CO and NO and the oxidized PHCP. B. Visible absorption spectra of the reduced PHCP wild‐type protein with butyl‐isocyanide and oxidized PHCP with imidazole. Specific wavelengths referred to in the text are indicated by arrows.

In order to confirm the ligand‐binding ability of the PHCP wild‐type, visible absorption spectra were obtained in the presence of CO, NO, butyl‐isocyanide, and imidazole [Fig. 7(A, B)]. The spectrum of the reduced PHCP with CO showed specific peaks at 418, 534, and 564 nm, consistent with the previous results for AVCP under the same temperature and pH conditions.^30^ As previously discussed, these specific peaks indicate that CO binds at the sixth position of the axial ligand of the heme. In contrast, the spectrum of the reduced PHCP with NO showed a specific peak at 395 nm, the 564 nm peak being increased and the 630 nm peak being decreased compared with oxidized PHCP without the NO binding [Fig. 7(A)]. These results are also consistent with the previous results for AVCP,^31^ suggesting that NO binds at the fifth position of the PHCP heme. The reduced state of PHCP in the presence of butyl‐isocyanide showed high specific peaks at 418, 527, and 557 nm [Fig. 7(B)], which was also confirmed for AVCP.^30^, ^32^ In addition, the spectrum of oxidized PHCP with imidazole showed specific peaks at 409 and 632 nm, which were already confirmed in *Rhodobacter capsulatus* cytochrome *c*′.^33^ Therefore, PHCP is able to bind CO, NO, and large ligands such as butyl‐isocyanide and imidazole, possibly through the channel, as discussed for AVCP.^22^

## Conclusions

In this study, we showed that the homo‐dimeric four‐helix bundle PHCP protein was stabilized by the presence of the heme, which interacted with amino acid side chains within a single subunit, as compared with the AVCP protein. This is consistent with the stability mechanism of monomeric globular class I cytochromes *c* from thermophiles.^11^, ^12^, ^13^, ^14^, ^15^, ^16^, ^17^ Interactions between heme and amino acid side chains are thus commonly responsible for the stability of thermophilic cytochromes *c*, regardless of their functions, whether electrons or larger ligands are relevant or not. In addition to the interaction within a single subunit of PHCP, the subunit–subunit interactions contributed more effectively to its high stability, which could never be achieved in monomeric class I cytochromes *c*.

Overall, it is concluded that dimeric PHCP is effectively stabilized through subunit–subunit interactions with conservation of the structure and ligand‐binding ability. This is because of the increasing stability because of the amino acid residues that do not form the ligand‐binding channel. The present study deepens the understanding of the stability and function of cytochromes *c*′. The need is indicated for future application studies on the PHCP protein aiming at the development of a stable sensor protein for physiologically important diatomic gasses such as NO and CO, as comprehensively reviewed recently.^18^

## Materials and Methods

### Crystallization

The authentic PHCP protein used for crystal structure determination was prepared from *H. thermoluteolus* cells, as described previously.^29^ The purified PHCP protein solution was dialyzed against pure water and then concentrated to ∼10 mg/mL. The initial crystallization conditions were screened by the hanging‐drop vapor diffusion method at 4°C using Crystal Screens I & II (Hampton Research, CA). Crystallization drops were prepared by mixing 2 μL of protein solution and 2 μL of reservoir solution, and were equilibrated against 500 μL of the reservoir solution. Initial crystals of the PHCP protein were grown using a reservoir solution consisting of 0.2*M* sodium acetate trihydrate, 30% (w/v) PEG 4,000, and 0.1*M* Tris‐HCl (pH 8.5). After several optimization steps, thin rod‐like crystals were obtained from a mixture of 2 μL of protein solution (10 mg/mL) and an equal volume of a reservoir solution consisting of 0.05*M* sodium citrate, 18% (w/v) PEG 4,000, and 0.1*M* Tris‐HCl (pH 8.1) at 4°C, within three days.

### X‐ray diffraction

The X‐ray diffraction experiment on a PHCP crystal was carried out on a Rigaku Micromax‐007 rotating anode X‐ray generator with a Cu target equipped with an R‐AXIS IV^++^ imaging plate detector (Rigaku, Japan). The crystal was flash‐cooled at −173°C in a stream of cold nitrogen, using following the cryoprotectant solution: 0.05*M* sodium citrate, 30% (w/v) PEG 4,000, and 0.1*M* Tris‐HCl (pH 8.1). A total of 270 images were collected for the PHCP crystal at a camera length of 150 mm and an oscillation angle of 1.0°. The PHCP crystal diffracted to a maximal resolution of 1.89 Å (Table 2). The diffraction data sets were processed using *CrystalClear* (Rigaku, Japan). The PHCP crystal belonged to the space group *P*2_1_22_1_ (*a* = 54.24 Å, *b* = 72.92 Å, *c* = 132.37 Å), and had four PHCP molecules in an asymmetric unit, with a Matthews coefficient of 2.15 Å^3^/Da, which corresponds to a solvent content of 42.7%.^34^

**Table 2:** Data Collection, Phasing, and Refinement Statistics for a PHCP Crystal

**Data collection**	
Wavelength (Å)	1.540
Resolution range (Å)^a^	43.52–1.89 (1.96–1.89)
Space group	*P*2_1_22_1_
Unit cell parameters	
*a*, *b*, *c* (Å)	54.24, 72.92, 132.37
*α*, *β*, *γ* (°)	90.0, 90.0, 90.0
Total measured reflections	401478
Unique reflections	41938
* R*_merge_ b	0.104 (0.342)
Average *I*/σ(/)	11.0 (3.8)
Completeness (%)	97.7 (83.6)
Wilson B‐factor (Å^2^)	17.4
**Refinement**	
Resolution (Å)	41.95–1.89 (1.93–1.89)
No. of reflections	41871
* R*_work_/*R*_free_ *^*c*^*	0.196/0.243
No. of atoms	5097
Water molecules	777
Overall B‐factors	18.62
R.m.s. deviations	
Bond length (Å)	0.012
Bond angle (°)	1.655
Ramachandran plot statistics	
Most favoured (%)	98.68
Disallowed (%)	0.00

a Values in parentheses are for the highest resolution shell.

b *R*_merge_ = ∑|*I*_h_ − <*I*_h_>|/∑*I*_h_, where <*I*_h_> is the average intensity of reflection h and symmetry‐related reflections. *^c^R*_work_ = ∑ǁ*F*_o_| − |*F*_c_ǁ/∑|*F*_o_|, where *F*_o_ and *F*_c_ are the observed and calculated structure factor amplitudes, respectively. *R*_free_ = *R*_work_ was calculated using 5% of the reflection data chosen randomly and set aside from the start of refinement.

### Molecular replacement and structure refinement

The structure of PHCP was determined by the molecular replacement method using program *Phaser* included in the PHENIX software suite,^35^ and the published crystal structure of AVCP (PDB ID: 1BBH) as a search model.^22^ The structural refinement, manual model building, and addition of water molecules were carried out using programs *phenix.refine* and *Coot*.^36^, ^37^ Finally, the refinement converged to an *R*_work_ of 0.196 and an *R*_free_ of 0.243. All of the residues in PHCP were successfully modeled, and the geometry of the final model of PHCP was checked with program *MolProbity*.^38^ The refinement statistics are listed in Table 2. The final coordinates have been deposited in the Protein Data Bank (PDB ID: 5B3I).

### Heterologous expression of the PHCP protein and its mutagenesis

The gene for the mature PHCP protein fused with the gene for the *Rhodopseudomonas palustris* cytochrome *c*_556_ signal peptide^39^ at the N‐terminus flanked by *Eco*RI and *Sal*I restriction sites was synthesized using favorable codons for *Escherichia coli* designed by a manufacturer (Operon, Japan). The resulting gene was inserted into the *Eco*RI and *Sal*I sites of the pKK223‐3 vector under the control of the *tac* promoter. The recombinant plasmid was transformed into *E. coli* strain JCB387 carrying the pEC86 plasmid encoding cytochrome *c* maturation proteins.^11^ The transformed *E. coli* cells were grown aerobically and a periplasmic protein fraction containing the heterologously expressed recombinant PHCP protein was extracted by the cold osmotic shock method.^40^ The resulting periplasmic protein fraction was used for purifying the recombinant PHCP protein, as described previously for the authentic protein.^29^ The concentration of the purified recombinant PHCP protein was determined with a Bradford protein assay kit (Bio‐Rad, Japan) using bovine serum albumin as a standard. The purified protein was subjected to protein sequencing analysis with an automatic protein sequencer (Shimadzu PPSQ‐31A, Japan).

Plasmids for expressing PHCP variants were prepared with Prime STAR Max DNA polymerase (TaKaRa Bio, Japan) and mutation primers (Supporting Information Table SI) using the wild‐type gene constructed in the pKK223‐3 vector as described above. Phe‐11, Thr‐17, Thr‐18, Ala‐20, Gln‐39, Phe‐71, and Leu‐76 in PHCP were separately substituted with Thr, Glu, Phe, Gly, Ala, Asp, and Val, respectively, which were the corresponding residues in AVCP (Fig. 2). Two triple variant genes carrying Thr‐17 to Glu, Ala‐20 to Gly, and Leu‐76 to Val substitutions located around the heme, and Phe‐11 to Thr, Thr‐18 to Phe, and Phe‐71 to Asp substitutions located on the subunit–subunit interface in the PHCP structure were also prepared by combination of single and double mutation primers. A sextuple variant gene carrying Phe‐11 to Thr, Thr‐17 to Glu, Thr‐18 to Phe, Ala‐20 to Gly, Phe‐71 to Asp, and Leu‐76 to Val substitutions at the same time was synthesized by a manufacturer (Eurofins Genomics, Japan) and ligated into the pKK223‐3 vector. The correct introduction of mutations was confirmed by DNA sequencing. All the resulting plasmids were used for the expression of PHCP variants in *E. coli* strain JCB387 as described for the wild‐type.^29^ Some of the resulting variant proteins were subjected to N‐terminal sequencing analysis, as described for the recombinant PHCP wild‐type protein.

### Thermal stability measurement

The thermal stabilities of the recombinant PHCP wild‐type and variants were measured by monitoring CD spectra using a JASCO J‐820 CD spectrometer (JASCO, Japan), as described previously.^6^ Protein solutions (20 μ*M*) dialyzed against 20 m*M* potassium phosphate (pH 7.0) were analyzed. The CD ellipticity change at 222 nm was monitored in a cuvette of a path length of 1 mm. CD values were recorded from 25°C to 100°C at intervals of 0.5°C and a heating rate of 1.0°C/min. The raw data were subjected to nonlinear least‐squares fitting, as described previously.^6^ Reversibility of protein denaturation was also examined as described previously.^6^

The data points obtained were corrected for the slope of the base lines for the native and denatured forms, and were normalized to calculate the fraction of the denatured protein. The resulting fraction was plotted as a function of temperature, and the resulting thermal denaturation curves were used to determine the thermodynamic parameters. The calculation was performed by curve fitting of the resulting CD values *versus* the temperature by van't Hoff analysis, and then the temperature at the midpoint of the transition (*T*_m_) and the enthalpy change during protein denaturation at *T*_m_ (Δ*H*) were determined. The entropy change during protein denaturation at *T*_m_ (Δ*S*) of the PHCP wild‐type protein was calculated with the equation Δ*S* = Δ*H*/*T*_m_. The fitting curves obtained in the temperature range of −40 to +40 of *T*_m_ were used to calculate thermodynamic parameters in order to reflect protein denaturation precisely. The differences in the free energy changes of denaturation between the variant proteins and the wild‐type at the wild‐type *T*_m_ (ΔΔ*G*_m_) were calculated using the equation given by Becktel and Schellman,^41^ ΔΔ*G*_m_ = Δ*T*_m_* Δ*S* (wild‐type), where Δ*T*_m_ is the difference in *T*_m_ value between the PHCP variant and wild‐type proteins, and Δ*S* (wild‐type) the entropy change of the wild‐type protein at *T*_m_.

### Analytical ultracentrifugation

Sedimentation velocity analysis of the recombinant PHCP wild‐type and variants was performed with a Beckman Optima XL‐I analytical ultracentrifuge (Beckman‐Coulter, CA) equipped with a 4‐hole An60 Ti rotor at 20°C using 12 mm double‐sector charcoal‐filled Epon centerpieces with quartz windows. Each protein solution (5 μ*M*) was dissolved in a buffer of 10 m*M* Tris‐HCl (pH 7.5) with 100 m*M* NaCl. Data were collected at 42,000 rpm using absorbance optics at 390 − 400 nm with a radial increment of 0.003 cm.

The distribution of sedimentation coefficients (*S*) was analyzed using the continuous *c*(*s*) distribution model in the program SEDFIT (version 14.1 Feb 2013).^42^ The positions of the meniscus and cell bottom were floated during the fitting. The range of sedimentation coefficients for fitting was 0–15 *S* with a resolution of 300. The frictional ratio was initialized at 1.2 and floated during the fitting. The partial specific volume of each protein, calculated from the amino acid composition, buffer density (*ρ*_0_ = 1.00260 g/cm^3^), and buffer viscosity (*η*_0_ = 0.01014 P), was estimated using the program SEDNTERP (http://sednterp.unh.edu).

### Ligand‐binding assay

The PHCP wild‐type protein (final 6 μ*M*) in 10 m*M* potassium phosphate buffer (pH 7.0) was air‐oxidized or reduced with a grain of sodium dithionite. The reduced PHCP protein solutions were bubbled with N_2_ (95.0%), and then with CO (99.9%, GL Science, Japan) in the dark. The same reduced PHCP solutions bubbled with N_2_ were loaded on a PD‐10 gel filtration column (GE Healthcare) equilibrated with 10 m*M* potassium phosphate buffer (pH 7.0) in order to remove excess sodium dithionite, and were then mixed with NONOate (diethylamine sodium salt hydrate, Sigma‐Aldrich; final 5 m*M*) in 10 m*M* NaOH (pH 8.0), which released NO gas under the neutral pH conditions, in the dark to determine whether or not the NO gas was bound. The air‐oxidized and reduced PHCP solutions were also mixed with 20 m*M* imidazole (Nacalai Tesque, Japan) and 0.5% (v/v) butyl‐isocyanide (Tokyo Chemical Industry, Japan), respectively. Visible absorption spectra of these preparations were obtained with a JASCO V‐530 spectrophotometer (JASCO, Japan) at 25°C.

## Conflict

The authors declare that they have no conflicts of interest with the contents of this article.

## Supporting information

Supporting Information
