# Supplementary material for: Structural and functional insights into thermally stable cytochrome c′ from a thermophile
Source: Protein Sci. 2017 Mar 6;26(4):737–48. doi: 10.1002/pro.3120 (PMC5368077; doi:10.1002/pro.3120)
Supplement: Supplementary file 1 — Supporting Information [file PRO-26-737-s001.docx]

**Supplementary materials**

**Structural and functional insights into thermally stable cytochrome *c*' from a thermophile**

Sotaro Fujii^1^, Hiroya Oki^2^, Kazuki Kawahara^2^, Daisuke Yamane^1^, Masaru Yamanaka^3^, Takahiro Maruno^4^, Yuji Kobayashi^4^, Misa Masanari^1^, Satoshi Wakai^5^, Hirofumi Nishihara^6^, Tadayasu Ohkubo^2^, and Yoshihiro Sambongi^1^*

^1^Graduate School of Biosphere Science, Hiroshima University, Higashi-Hiroshima, Hiroshima, Japan, ^2^Graduate School of Pharmaceutical Sciences, Osaka University, Yamadaoka, Suita, Osaka, Japan, ^3^Graduate School of Materials Science, Nara Institute of Science and Technology, Ikoma, Nara, Japan, ^4^Graduate School of Engineering, Osaka University, Yamadaoka, Suita, Osaka, Japan, ^5^Graduate School of Science, Technology, and Innovation, Kobe University, Rokkodai, Kobe, Hyogo, Japan, and ^6^Faculty of Agriculture, Ibaraki University, Inashikigun, Ibaraki, Japan

**Contents**

Supplemental Table: SI

Supplemental Figures: S1 – S7

**Supplemental Table SI.** *Sequences of oligonucleotide primers used for mutagenesis.*

Underlined nucleic acid residues are those that were mutated.

|  | PHCP variants |  | Primer | Sequence (5' to 3') |
| --- | --- | --- | --- | --- |
|  | F11T |  | Fw | GTCAAAACCCGCCAAGCGTCGTACACC |
|  |  |  | Rv | TTGGCGGGTTTTGACCTTGTCCTCCGG |
|  | T17E |  | Fw | TCGTACGAAACCATGGCCTGGAATATG |
|  |  |  | Rv | CATGGTTTCGTACGACGCTTGGCGAAA |
|  | T18F |  | Fw | TACACCTTTATGGCCTGGAACATGGGC |
|  |  |  | Rv | GGCCATAAAGGTGTACGACGCTTGGCG |
|  | A20G |  | Fw | ACCATGGGCTGGAACATGGGCAAAATC |
|  |  |  | Rv | GTTCCAGCCCATGGTGGTGTAGCTGGC |
|  | Q39A |  | Fw | TTCAGCGCAACGCAGGTCTCTGCAGCC |
|  |  |  | Rv | CTGCGTTGCGCTGAACGGCATCGTGCC |
|  | F71D |  | Fw | GTGGGGGATAAGAAAAGCCGTCTGAAG |
|  |  |  | Rv | TTTCTTATCCCCCACTACTCCCAAGGT |
|  | L76V |  | Fw | AGCCGTGTGAAGGAGAACTTCTTTCAG |
|  |  |  | Rv | CTCCTTCACACGGCTTTTCTTGAACCC |
|  | T17E/A20G |  | Fw | TCGTACGAAACCATGGGCTGGAATATG |
|  |  |  | Rv | CATGGTTTCGTACGACGCTTGGCGAAA |


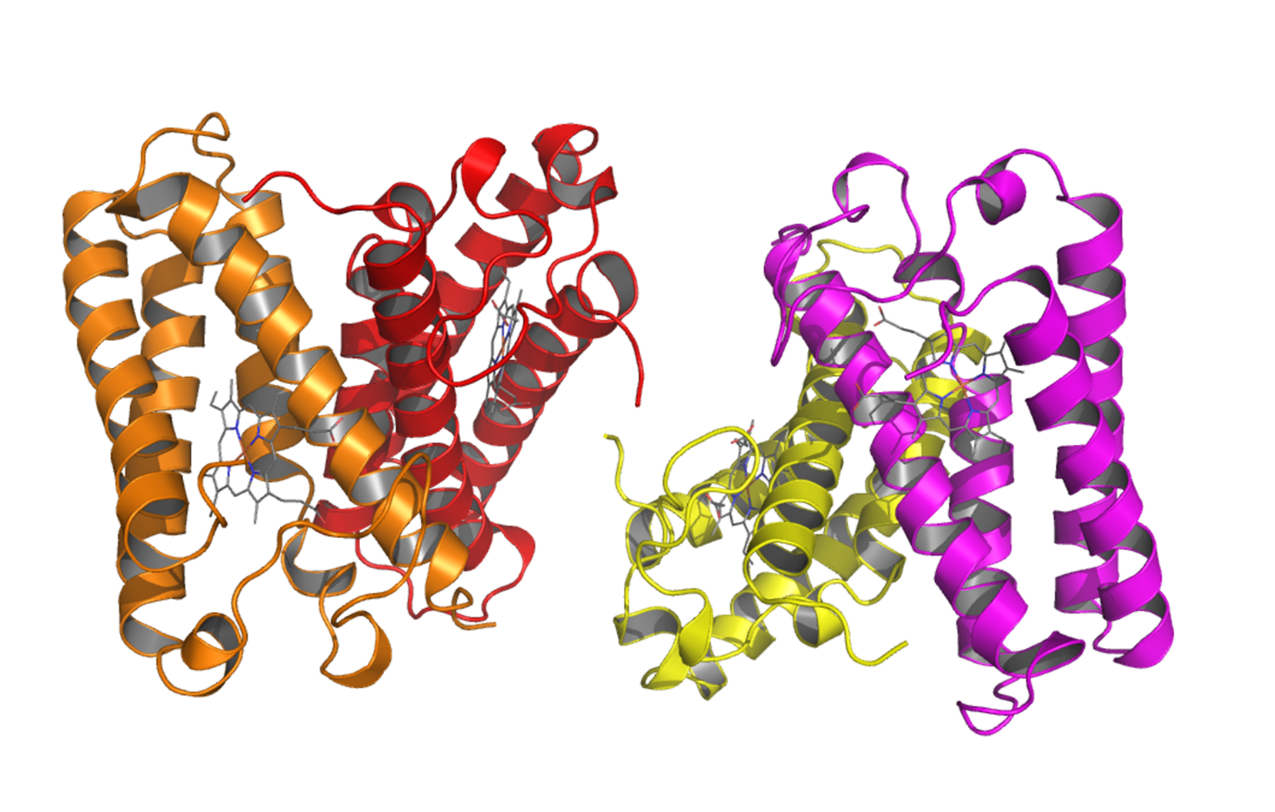


**Supplemental Figure S1.**  Overall structure of the two dimeric forms in the PHCP crystal. The main chain and heme are presented as ribbon and stick models, respectively. Single subunits are colored red, orange, yellow and magenta, respectively.


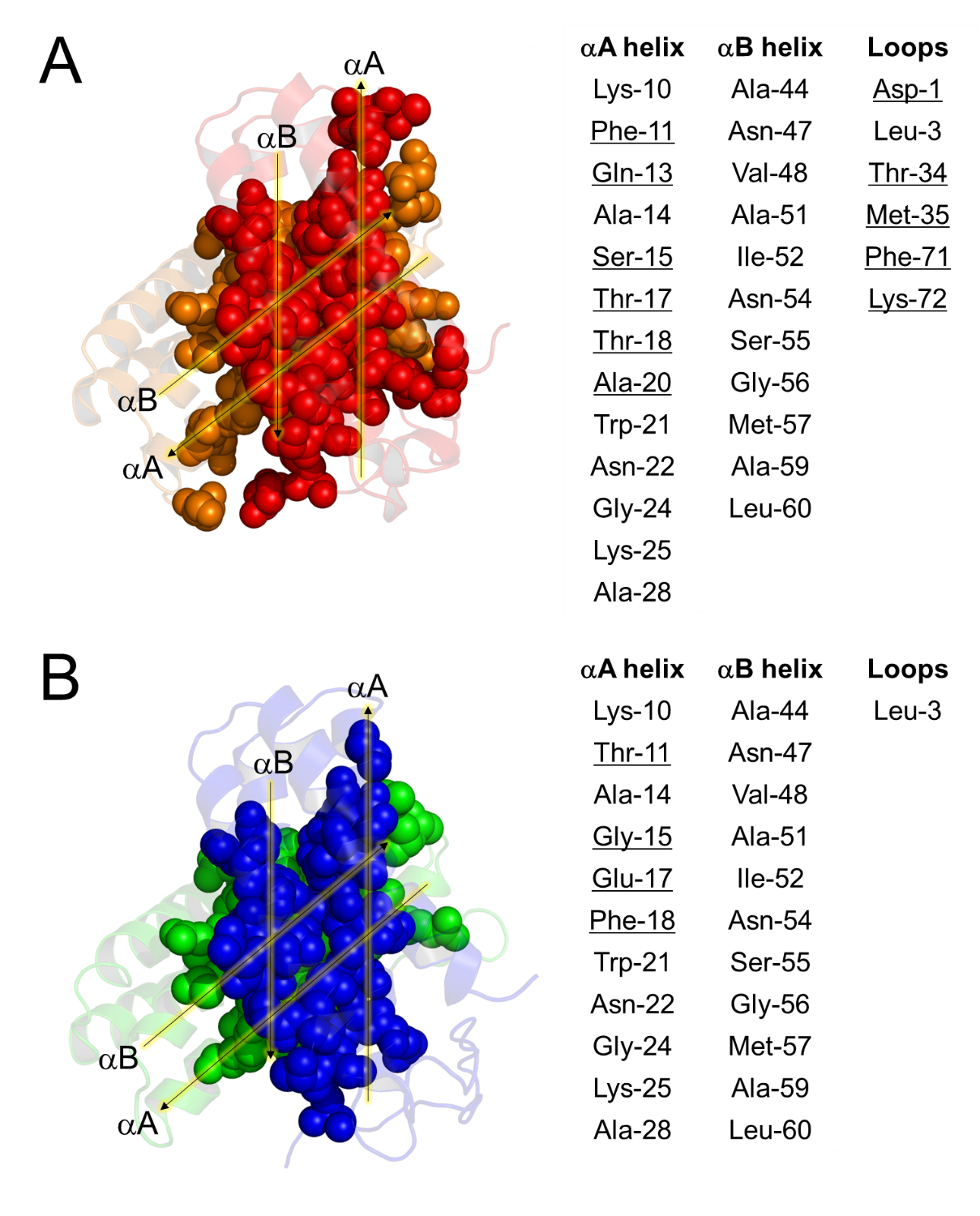


**Supplemental Figure S2.**  A. The subunit-subunit interface of PHCP. The listed amino acid residues on the subunit-subunit interface are shown as red and orange spheres. B. The subunit-subunit interface of AVCP. The amino acid residues on the subunit-subunit interface are shown as blue and green spheres. The locations of αA and αB helices are indicated by arrows. The different amino acid residues on the interfaces between PHCP and AVCP are underlined.


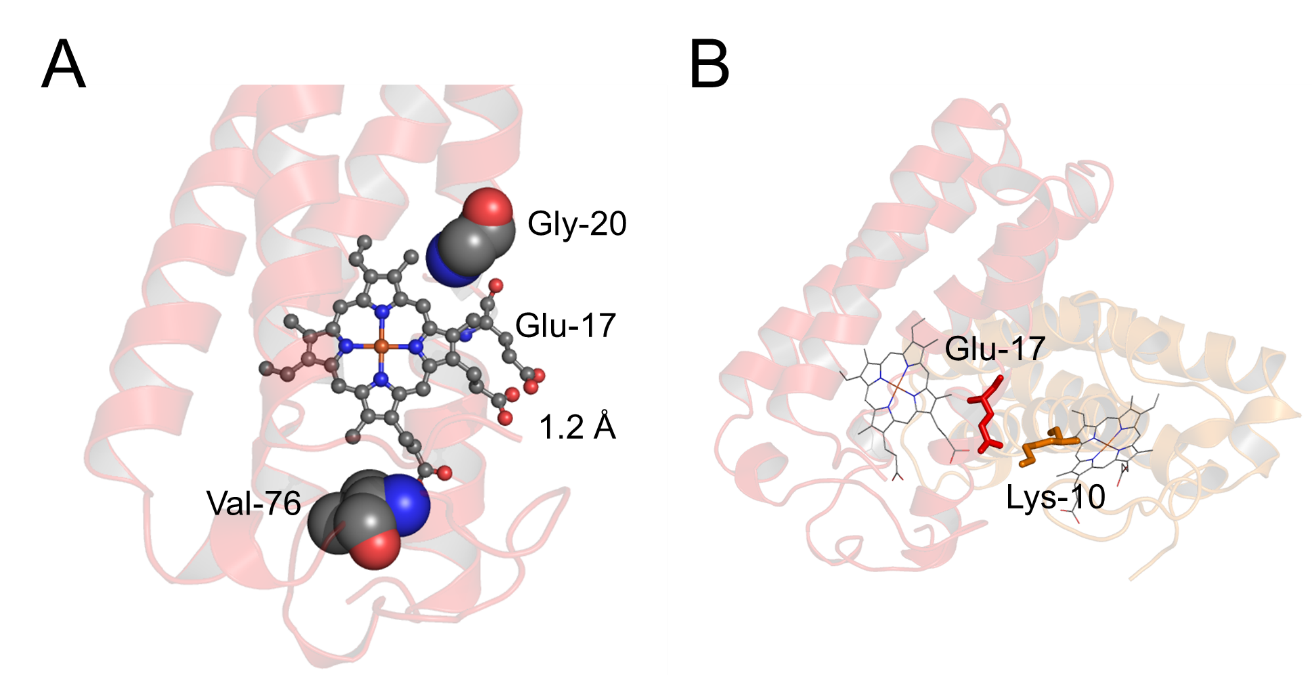


**Supplemental Figure S3.**  Structure simulation for the PHCP variants using the Pymol program: Altered heme-related interactions. A. Glu-17 introduced resulted in steric hindrance with the heme. Gly-20 and Val-76 introduced resulted in a void space. B. Glu-17 introduced may form a salt bridge with Lys-10 in the other subunit.


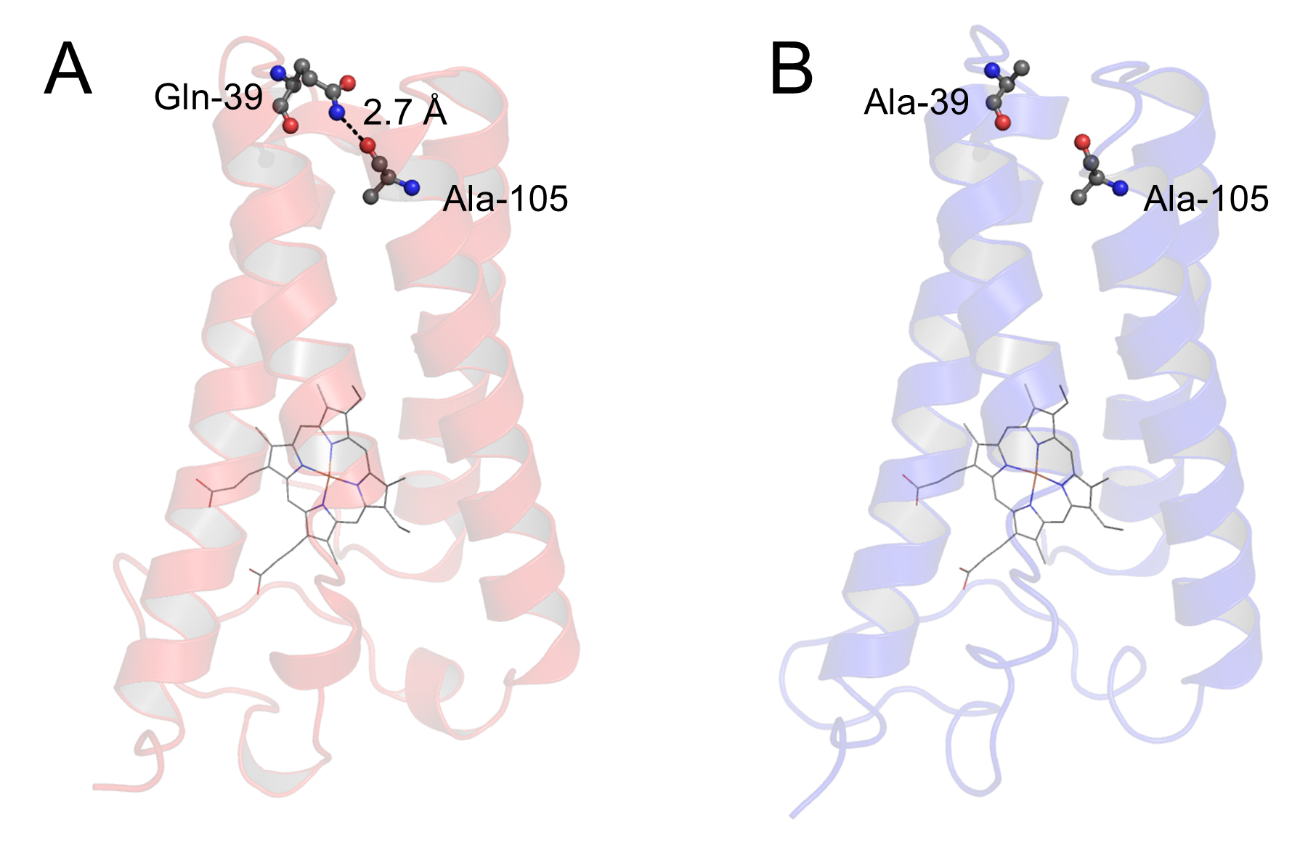


**Supplemental Figure S4.** Interactions on a single subunit surface. A. PHCP structure. B. The corresponding AVCP structure. The main chains and hemes are presented as ribbon and ball-and-stick models, respectively. Specific amino acid residues are also presented as stick models. The hydrogen bond is shown as a black dotted line.


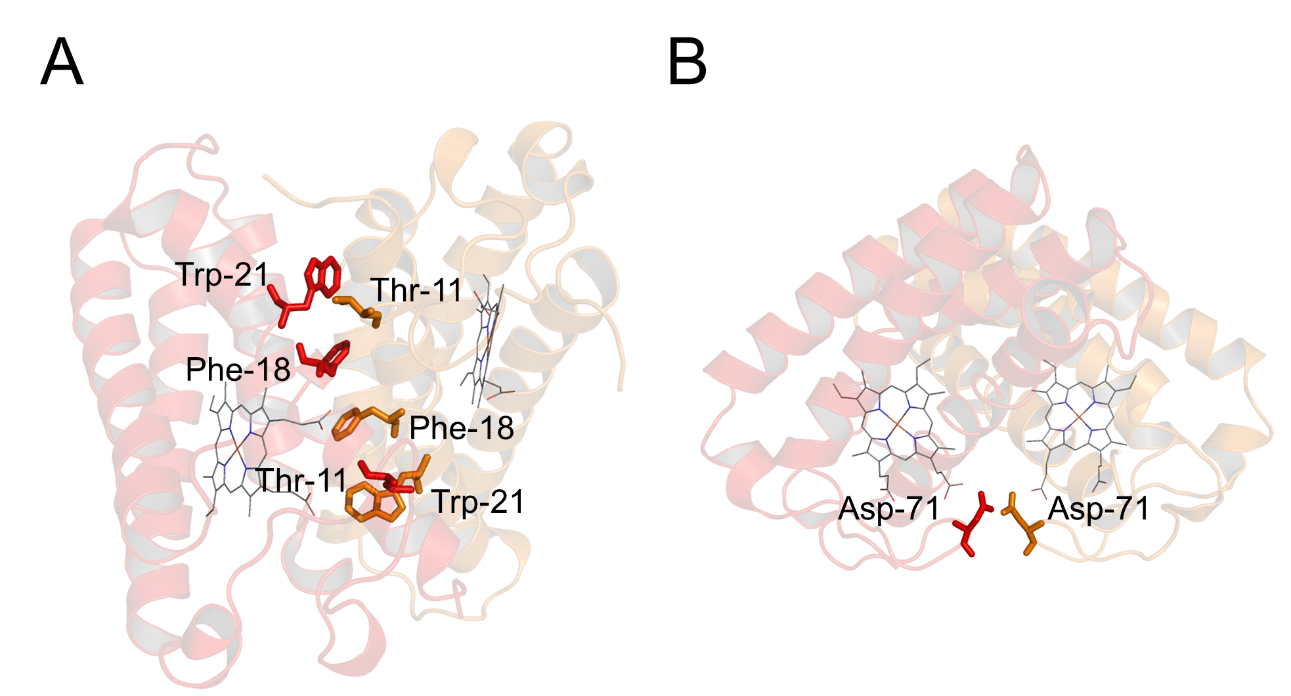


**Supplemental Figure S5.** Structure simulation for the PHCP variants using the Pymol program: Altered subunit-subunit interactions. A. Thr-11 faced Trp-21 without a specific interaction. Phe-18 hydrophobically interacted with surrounding residues, but not with a hydrogen bond, as observed for the original Thr-18 residue. B. Asp-71 introduced caused destabilization in the local hydrophobic environment.


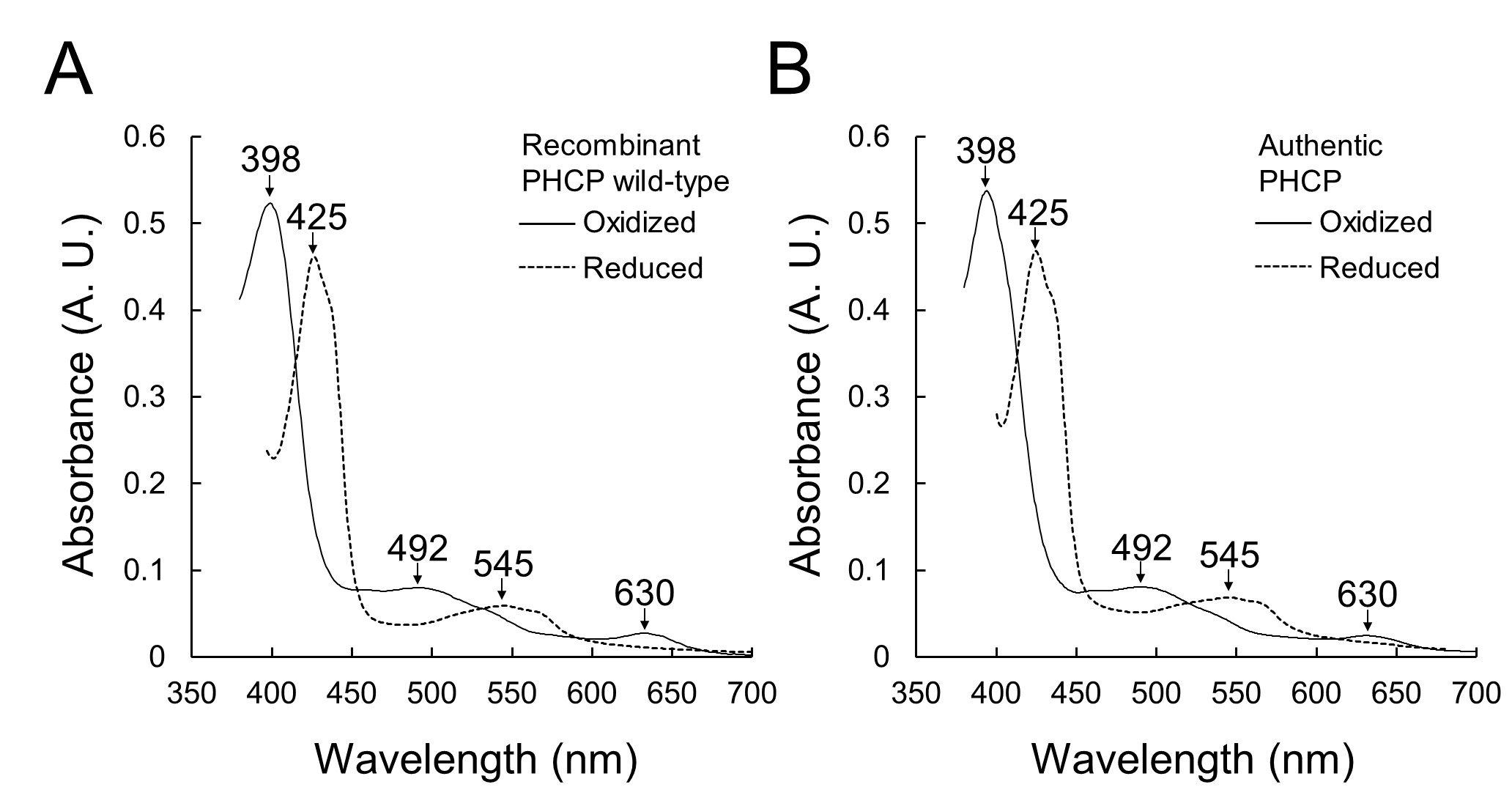


**Supplemental Figure S6.**  Visible absorption spectra of oxidized and reduced PHCP. A. Recombinant PHCP wild-type expressed in *E. coli* cells. B. Authentic PHCP purified from *H. thermoluteolus* cells.^29^ Specific peaks are indicated by arrows.


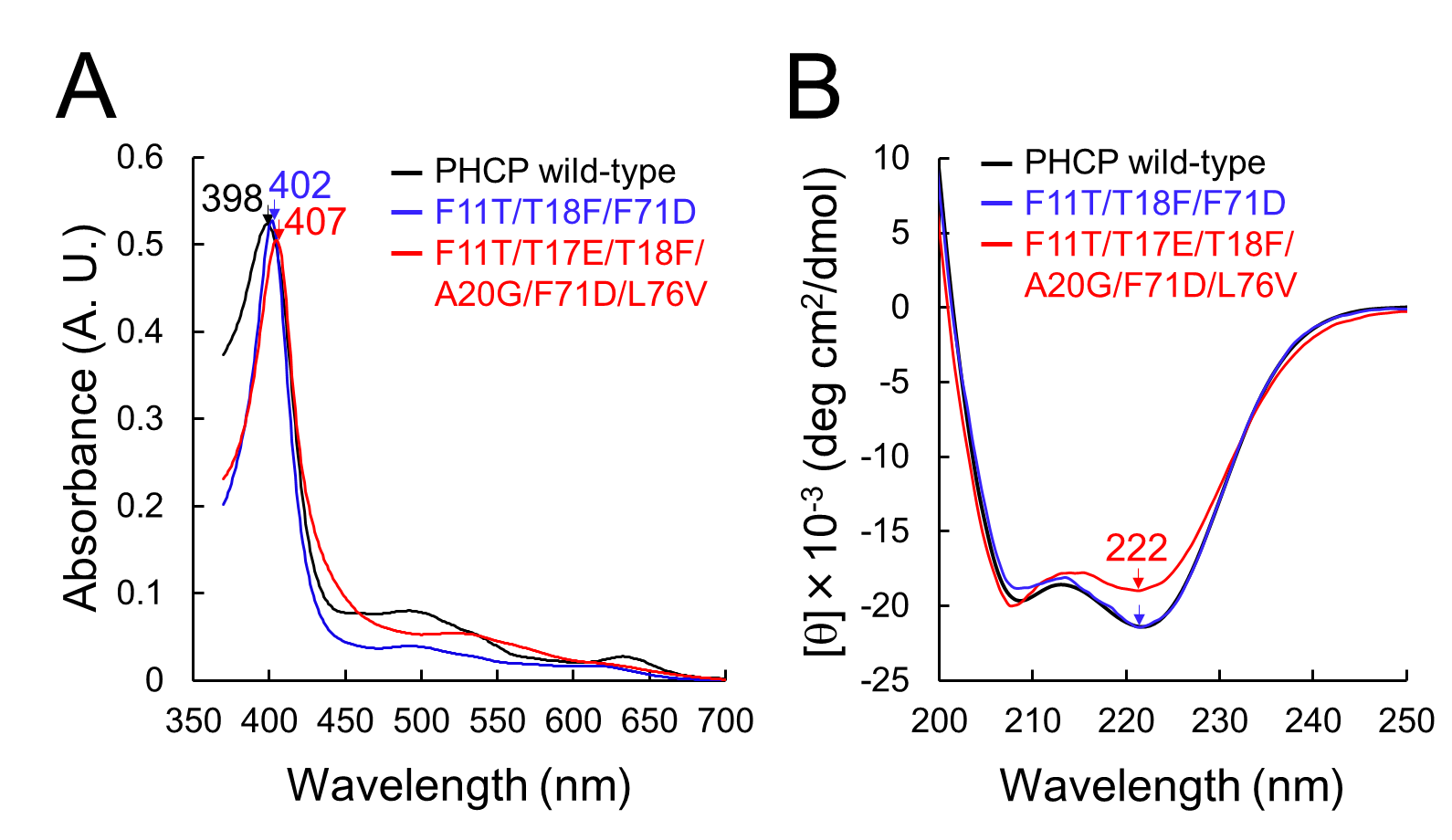


**Supplemental Figure S7**. A. Visible absorption spectra of oxidized PHCP wild-type and multiple variants. B. CD spectra of the PHCP wild-type and multiple variants. Specific wavelengths referred to in the text are indicated by arrows.
